# Walking Aids and Locomotion Training in the Emergency Department: A Randomized Clinical Trial

**DOI:** 10.1001/jamanetworkopen.2025.44535

**Published:** 2025-11-21

**Authors:** Fernanda Sato Polesel, Sâmia Denadai, Christian Valle Morinaga, Itiana Cardoso Madalena, Marlon Juliano Romero Aliberti, Expedita Angela Henrique, Mario Chueire de Andrade-Junior, Ricardo Aparecido Baptista Nucci, Wellington Pereira Yamaguti, Pedro Kallas Curiati, Renato Fraga Righetti

**Affiliations:** 1 Geriatric Emergency Department Research Group, Hospital Sírio-Libanês, São Paulo, Brazil; 2 Rehabilitation Service, Hospital Sírio-Libanês, São Paulo, Brazil; 3 Geriatric Center for Advanced Medicine, Hospital Sírio-Libanês, São Paulo, Brazil; 4 Teaching and Research Institute, Hospital Sírio-Libanês, São Paulo, Brazil

## Abstract

**Importance:**

Mobility limitations are common in older adults and impact quality of life and social interaction. Walking aids can improve mobility and prevent falls but require oversight and training. Despite being recommended by geriatric emergency department (ED) guidelines, their effectiveness in this setting has not been studied to date.

**Objective:**

To evaluate the effectiveness of training and provision of walking aids, with or without telemonitoring, on mobility, fear of falling, gait, functional capacity, quality of life, cognition, depression, and occurrence of falls in older adults after an ED visit compared with safe ambulation recommendations only.

**Design, Setting, and Participants:**

This randomized clinical trial was conducted at the Geriatric ED of Hospital Sírio-Libanês, São Paulo, Brazil, from July 20 to December 16, 2023, with a 90-day follow-up. Participants were patients 65 years or older who were discharged from the geriatric ED with at least 1 indication for walking aids according to the institutional protocol. Researchers involved in postintervention assessment and statistical analyses were blinded to group allocation.

**Intervention:**

Seventy-five participants were allocated to 1 of 3 groups: control, walking aids (WA), or walking aids with telemonitoring (WAT). All participants received safe ambulation recommendations. Intervention groups (WA and WAT) were assessed and trained in device use by a physiotherapist. The WAT group received telemonitoring follow-up to promote adherence.

**Main Outcomes and Measures:**

The primary outcomes were improved life-space mobility as assessed by the Life Space Assessment and fear of falling as assessed by the Falls Efficacy Scale International at 90 days follow-up. Secondary outcomes included gait, functional capacity, quality of life, cognition, depression, and occurrence of falls.

**Results:**

A total of 75 older adults were enrolled (mean [SD] age, 81.3 [7.7] years; 40 [53.3%] female). At 90 days, the WA group had significant improvement in life-space mobility (mean difference [MD], 12.77; 95% CI, 1.06-24.54; *P* = .03), fear of falling (MD, –5.60; 95% CI, –9.06 to –2.14; *P* = .002), and 1-minute sit-to-stand test (MD, 8.45; 95% CI, 4.34-12.56; *P* = .001) compared with the control group. No significant improvements were observed in any of these outcomes in the WAT group compared with the WA group.

**Conclusions and Relevance:**

In this randomized clinical trial, training and provision of walking aids for older adults in the ED improved mobility and fear of falling as long as 90 days after discharge. The addition of telemonitoring did not result in additional benefits. This study highlights the role of a specialized physiotherapy intervention to optimize outcomes in older adults in the ED.

**Trial Registration:**

ClinicalTrials.gov Identifier: NCT05950269

## Key Points

**Question:**

Does a program of training and provision of walking aids in the emergency department, with or without telemonitoring, improve mobility in life spaces and fear of falling in older adults after 90 days?

**Findings:**

In this randomized clinical trial of 75 adults 65 years or older, the intervention groups had a significant improvement in mobility and fear of falling at 90 days compared with the control group. Telemonitoring did not improve mobility compared with no telemonitoring in the intervention groups.

**Meaning:**

A program of training and provision of walking aids improved mobility and fear of falling among older adults discharged from the emergency department.

## Teaser

This randomized clinical trial evaluates the effectiveness of training and provision of walking aids delivered by physiotherapists, with or without telemonitoring, on mobility among older adults in the emergency department.

This randomized clinical trial evaluates the effectiveness of training and provision of walking aids delivered by physiotherapists, with or without telemonitoring, on mobility and fear of falling among older adults in the emergency department.

## Introduction

The global population is aging rapidly, with projections indicating that by 2050, 2.1 billion people will be 60 years or older.^1^ In 2021, patients 65 years and older accounted for 19.4% of emergency department (ED) visits in the US and were more likely to be hospitalized than younger patients.^2^ This demographic shift poses substantial challenges for health care systems, particularly in the ED, and older adults often face a higher risk of adverse outcomes due to atypical disease presentations, polypharmacy, comorbidities, and functional limitations.^3,4^ Nevertheless, the ED plays a fundamental role for them: 24-hour accessible medical care, rapid assessment, stabilization and referral for hospital admission, referral to community resources, emergency treatment, and entry point for high-complexity acute care.^5,6,7^ Several guidelines recommend mobility assessment before ED discharge for older patients.^4^ They also highlight the importance of specialized protocols and procedures for these patients in EDs, covering the Geriatric 5Ms framework: mentation, mobility, medications, multicomplexity, and matters most.^5,8,9^

Mobility refers to the physical ability to move and adapt to the environment, including walking, climbing stairs, and transferring with or without assistance. Functional capacity refers to an individual’s ability to perform activities that are necessary or desirable in their lives, whether at work, in daily life, or in recreational settings. Mobility is linked to independence, autonomy, and quality of life.^10,11^ Walking aids can improve balance, stability, and confidence; reduce fall risk; and promote autonomy.^12,13^ Furthermore, prescription of walking aids and training by a qualified professional, such as a physiotherapist, is crucial to optimize their benefits and minimize potential risks.^12,13^

In addition, telemonitoring is a health care intervention that has been growing and holds great promise for improving care for older adults. It is defined as the use of information technology and telecommunications to deliver health care remotely.^14^ Benefits include reducing unnecessary clinic and home visits, minimizing infection risks, facilitating rehabilitation monitoring, and improving patient adherence.^15^

Although walking aids have been a prerequisite for Geriatric Emergency Department Accreditation since 2018,^16^ their use in this setting is mainly based on expert opinion. Moreover, guidelines recommend a multidisciplinary staff focused on assessing the needs of the geriatric population, and physiotherapists are essential to this team, guiding and training patients regarding functional needs.^5,16^ Therefore, the goal of this study was to evaluate the effectiveness of training and provision of walking aids delivered by physiotherapists—with or without telemonitoring—on mobility, fear of falling, functional capacity, quality of life, cognition, depression, and occurrence of falls in older adults in the ED. In addition, a telemonitoring intervention was incorporated to reinforce guidance on safe ambulation and proper use of the walking aid, thereby supporting adherence.

## Methods

### Study Design

A full description of the study protocol for this randomized clinical trial was published previously^17^ and is also available in Supplement 1. The trial was conducted at the geriatric ED of Hospital Sírio-Libanês (HSL), a tertiary hospital in São Paulo, Brazil, from July 20 to December 16, 2023. HSL received Geriatric Emergency Department Accreditation in 2019^16^ and provides specialized care for older adults, including comprehensive geriatric assessment and access to multidisciplinary teams. This study was approved by the Ethics Committee of HSL and followed the Consolidated Standards of Reporting Trials (CONSORT) reporting guideline. All patients provided written informed consent.

### Participants and Setting

We included patients 65 years and older who were discharged from our geriatric ED and met at least 1 indication for walking aids according to our institutional balance and gait protocol implemented by a physiotherapist in the ED. Possible indications consisted of reduction of postural instability, improvement of motor control, increase of somatosensory feedback, reduction of biomechanical overload, safe promotion of autonomy, and fall history (in the last 6 months).^18^

Exclusion criteria encompassed altered level of consciousness, need for supplemental oxygen (≥3 L/min), respiratory distress, hemodynamic instability, postural instability with a tendency to fall backward, cognitive impairment that limits the use of walking aids, hospitalization following the ED visit, and delirium. Delirium was assessed using the Confusion Assessment Method.^19,20,21,22^ Acquisition of informed consent, randomization, and data collection were performed by research assistants (including E.A.H.) with Research Electronic Data Capture (REDCap) software.^23^

### Interventions

After randomization, interventions were implemented across 3 groups: control, walking aids training (WA), and walking aids training with telemonitoring (WAT). All participants received recommendations for safe ambulation^22^ and a printed leaflet with relevant information^17^ (eMethods 1 in Supplement 2). Participants in the intervention groups were assessed on their mobility needs and trained in the use of the device deemed appropriate (cane or walker) by a physiotherapist^17^ (eMethods 2 in Supplement 2). Additionally, participants in the WAT group were followed up twice weekly during 3 months with telemonitoring (via video call) to promote and reinforce the importance of walking aids and safe ambulation. Telemonitoring was conducted by a nurse (E.A.H. and S.D.). The control group received only verbal instructions and printed materials containing guidelines for safe ambulation (eMethods 1 in Supplement 2).

### Outcomes

Between-group comparisons were conducted at 90 days for the WA vs control groups. In addition, an exploratory analysis was performed including the comparison between the WAT vs WA groups. Intragroup changes from baseline to 90 days were also evaluated. All analyses were performed for both primary and secondary outcomes.

#### Primary Outcomes

##### Mobility

Mobility was assessed using the Life-Space Assessment (LSA), a validated self-reported tool that captures life-space mobility during the previous 4 weeks.^24,25,26,27^ It estimates the extent (from within the home to beyond the city), frequency, and level of independence of an individual’s movement. LSA scores range from 0 (bedroom-bound) to 120 (frequent independent travel beyond the city).^24,25,26,27^ The LSA assesses mobility and is a strong inverse predictive factor associated with adverse outcomes in older adults, including falls, cognitive decline, hospitalization, institutionalization, and death.^26,27^ It also incorporates key factors such as use of assistive devices, social engagement, cognitive health, and mental well-being.^26,27^

##### Fear of Falling

Fear of falling is defined as a prolonged concern about falling that may result in individuals avoiding daily activities.^28^ Fear of falling was evaluated by the Falls Efficacy Scale International (FES-I),^29,30^ which consists of daily activities and postural control using 16 items, each rated on a 4-point scale. Total scores range from 16 to 64, with higher scores indicating worse levels of efficacy and control.^29,30^

#### Secondary Outcomes

##### Functional Capacity

The functionality assessment consisted of 4 measures: 1-minute sit-to-stand test,^31,32^ Katz Index,^33,34^ Barthel Index,^35,36,37,38^ and Lawton-Brody Scale.^39^ In the 1-minute sit-to-stand test, participants were asked to stand up and sit down completely as many times as possible in 1 minute.^39^ The Katz Index was used to evaluate 6 activities related to self-care and scored patients from 0 (independence) to 6 (total dependence).^33,34^ The Barthel Index also evaluates basic activities of daily living (ADL), focusing on self-care and mobility, with higher scores (80-100) indicating greater independence.^35,36,37,38^ Finally, the Lawton-Brody Scale assesses independence in 9 instrumental ADL, including telephone, transportation, finances, and medication, with scores ranging from 9 (totally dependent) to 27 (independent).^39^

##### Gait

Gait was assessed using the Timed Up and Go (TUG) test,^40^ which evaluates fall risk and functional mobility, strength, agility and balance.^41,42^ Participants were instructed to stand up from the chair, walk 3 m, go around a cone, and then return to the chair and sit down.^43^ A mean time was calculated from 3 trials. At baseline, participants performed the test without walking aids, and after the intervention, the test was repeated using the walking aid in the intervention groups.

##### Quality of Life

Quality of life was assessed using Euro Quality of Life Instrument, Five-Dimensions, Three-Level (EQ-5D-3L), which explores 5 dimensions of health: mobility, self-care, usual activities, pain and/or discomfort, and anxiety and/or depression.^44,45,46^ Each dimension is classified into 3 levels: no problems, some problems, and extreme problems. In addition, the instrument also has a self-assessment of health using a visual analog scale ranging from 0 (the worst health you can imagine) to 100 (the best health you can imagine).^44,45,46^

##### Cognition

Cognition was assessed using the 10-Point Cognitive Screener (10-CS), a brief 2-minute tool that evaluates orientation, memory recall, and verbal fluency. It requires no motor skills or writing, making it well-suited for use in ED settings.^47,48^ Final scores, adjusted according to educational level, can indicate normal cognition (≥8 points), possible cognitive impairment (6-7 points), or probable cognitive impairment (0-5 points).^47,48,49^

##### Depression

The 15-item Geriatric Depression Scale (GDS-15) was used to assess depression. This tool contains 15 items with a dichotomous (yes or no) response option. A total score higher than 5 indicates clinically significant symptoms; 6 to 10, mild depression; and 11 to 15, severe depression.^50^

##### Occurrence of Falls

The occurrence of falls was monitored during the whole study period. Participants were asked to complete a diary to record each fall (including location, associated injuries, and need for special care), as well as the total number of falls. This information was retrieved at the 90-day follow-up interview.

### Assessment Time Points

Baseline assessments were conducted before randomization and included sociodemographic and clinical variables and geriatric vulnerability using the PRO-AGE score (physical impairment, recent hospitalization, older age [≥90 years], acute mental alteration, getting thinner, and exhaustion), a validated tool for older adults in the ED.^51^ Geriatric vulnerability was classified based on risk of hospital admission or prolonged stay.^51^ Functional and clinical measures (eg, LSA, 1-minute sit-to-stand test, Katz Index, Barthel Index, Lawton-Brody Scale, TUG test, FES-I, EQ-5D-3L, 10-CS, and GDS-15) were collected without walking aids. Immediately after the intervention or after receiving safe ambulation recommendations, the TUG test and FES-I were repeated in the ED with walking aids in the WA and WAT groups and without walking aids in the control group. At the 90-day follow-up, conducted by a physiotherapist (F.S.P.) via phone or video call, all assessments (except the TUG test) were repeated. Fall events and walking aid use at the time of falls were also recorded.

### Sample Size

Sample size calculation was based on the findings of Kennedy et al,^51^ who reported a minimum clinically important difference of 5 points on the LSA scale (intragroup change), with an SD of 5.1 points. The total sample size needed was determined to be 66 individuals (adjusted for a 3-arm study) for an α of .05 and a power of 0.80. Estimating a 15% loss to follow-up, the sample size needed for the study’s success was increased to 75 individuals, randomized (1:1:1) into 25 participants in each group.

### Randomization and Blinding

Participants were randomized in a 1:1:1 ratio using computer-generated block randomization (block size = 9) implemented in REDCap. Allocation was fully automated, with no researcher involvement. Outcome assessors and the statistician (R.F.R.) remained blinded to group assignment throughout the study.

### Statistical Analyses

Continuous outcomes were analyzed using linear regression models adjusted for baseline values, and results were reported as mean differences (MD) with 95% CIs for the between-group comparisons at 90 days for WA vs control groups and, in the exploratory analysis, for the WA vs WAT groups. Additionally, linear regression models were used to assess intragroup changes from baseline to 90 days for both primary and secondary outcomes, with results expressed as MDs and 95% CIs. For the occurrence of falls, Fisher exact test was used. The analysis of the EQ-5D-3L dimensions used a 2-proportions test. Missing data were handled using multiple imputation by chained equations under the missing-at-random assumption. The imputed datasets were analyzed separately, and results were combined according to the Rubin rules to obtain valid statistical inferences. A total of 3 imputations were performed in the WA group, 6 in the WAT group, and 7 in the control group, restricted to the 90-day follow-up period.

All analyses were performed using SPSS, version 28.0.1 (IBM Corporation). Statistical significance was defined as 2-tailed *P* < .05.

## Results

### Participant Characteristics

A total of 424 potentially eligible individuals were identified, of whom 75 were enrolled (25 per group). The mean (SD) age was 81.3 (7.7) years; 40 participants (53.3%) were female and 35 (46.7%) were male. The median handgrip strength was 10.0 (IQR, 5.2-9.7) kg-F, and 19 participants (25.3%) were dependent for self-care. The most frequent indications for protocol inclusion were reduction of postural instability (71 [94.7%]), safe promotion of autonomy (46 [61.3%]), and history of falls (35 [46.7%]). Table 1 presents baseline participant characteristics. Seven participants were lost to follow-up in the control group, 3 in the WAT group, and 6 in the WAT group (Figure).

**Table 1.** Baseline Characteristics of Participants

Characteristic	Participant group, No. (%)			
	All (N = 75)	Control (n = 25)	WA (n = 25)	WAT (n = 25)
Age, mean (SD), y	81.3 (7.7)	81.9 (9.1)	79.9 (6.9)	82.2 (7.0)
Sex				
Female	40 (53.3)	14 (56.0)	14 (56.0)	12 (48.0)
Male	35 (46.7)	11 (44.0)	11 (44.0)	13 (52.0)
BMI, mean (SD)	26.3 (4.0)	25.4 (4.3)	27.4 (3.4)	26.2 (4.1)
Calf circumference, mean (SD), cm	31.69 (5.52)	31.44 (5.41)	32.40 (5.68)	31.24 (5.62)
Handgrip strength, median (IQR), kg-F	10 (5.2-9.7)	8.0 (4.0-20.0)	11.0 (8.8-19.0)	11.0 (5.0-19.0)
Dependency for self-care	19 (25.3)	6 (24.0)	6 (24.0)	7 (28.0)
Home adaptations for fall prevention	60 (80.0)	21 (84.0)	21 (84.0)	18 (72.0)
Geriatric vulnerability (PRO-AGE score, prolonged length of stay model), median (IQR)^a^	2 (1-4)	2 (1-4)	2 (1-3)	3 (1-4)
Comorbidities				
Alcoholism	1 (1.3)	1 (4.0)	0	0
Dementia	1 (1.3)	0	1 (4.0)	0
Depression	9 (12.0)	5 (20.0)	3 (12.0)	1 (4.0)
Neurologic disorder	5 (6.7)	2 (8.0)	2 (8.0)	1 (4.0)
Rheumatic diseases	2 (2.7)	0	0	2 (8.0)
Educational level				
Completed high school	11 (14.7)	2 (8.0)	3 (12.0)	6 (24.0)
Did not complete high school	11 (14.7)	4 (16.0)	2 (8.0)	5 (20.0)
Complete ungraduate degree	46 (61.3)	16 (64.0)	17 (68.0)	13 (52.0)
Postgraduate	7 (9.3)	3 (12.0)	3 (12.0)	1 (4.0)
Living alone	24 (32.0)	7 (28.0)	9 (36.0)	8 (32.0)
Indication for protocol				
Reduction of postural instability	71 (94.7)	22 (88.0)	24 (96.0)	25 (100)
Increase of somatosensory feedback	1 (1.3)	1 (4.0)	0	0
Improvement of motor control	12 (16.0)	2 (8.0)	6 (24.0)	4 (16.0)
Reduction of biomechanical overload	5 (6.7)	0	2 (8.0)	3 (12.0)
Safe promotion of autonomy	46 (61.3)	14 (56.0)	16 (64.0)	16 (64.0)
Fall history (in the last 6 mo)	35 (46.7)	13 (52.0)	12 (48.0)	10 (40.0)
Clinical Frailty Scale				
Median (IQR)^b^	4 (4-5)	4 (4-5)	4 (4-5)	5 (4-5)
Vulnerable	32 (42.7)	14 (56.0)	9 (36.0)	9 (36.0)
Mildly frail	20 (26.7)	4 (16.0)	6 (24.0)	10 (40.0)
National Early Warning Score, median (IQR)^c^	1 (0-2)	1 (1-2)	1 (0-2)	1 (1-2)

Abbreviations: BMI, body mass index (calculated as weight in kilograms divided by height in meters squared); PRO-AGE, physical impairment, recent hospitalization, older age (≥90 years), acute mental alteration, getting thinner, and exhaustion; WA, walking aid; WAT, walking aid with telemonitoring.

^a^ Scores range from 0 to 8, with higher scores indicating greater vulnerability.

^b^ Scores range from 1 to 9, with higher scores indicating greater frailty.

^c^ Scores range from 0 (normal) to 3 (at risk) for vital signs and other assessments.

**Figure.**
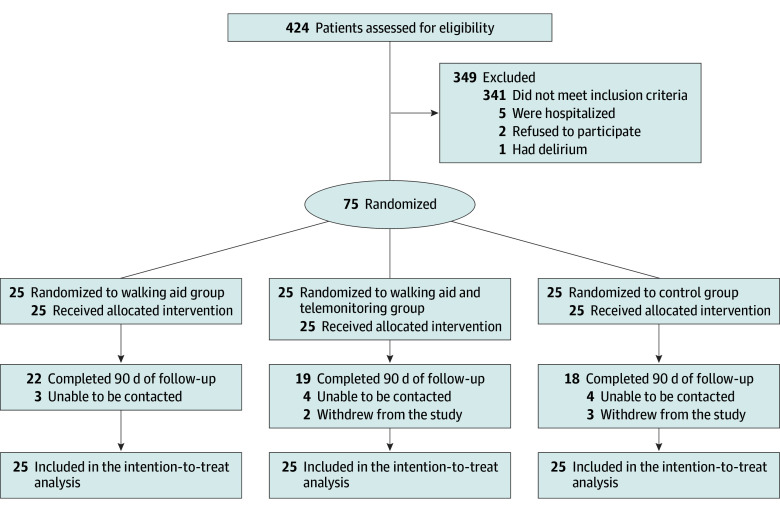
Flow Diagram of Participants

### Primary Outcomes

#### Mobility

At 90 days, the WA group had a statistically significant increase in LSA score compared with the control group (MD, 12.77; 95% CI, 1.06-24.54; *P* = .03). No significant difference was observed between WAT and WA groups at 90 days (MD, 1.52; 95% CI, −9.59 to 12.83; *P* = .78) (Table 2). Within-group analyses showed a significant improvement in LSA score from baseline to 90 days for both the WA (MD, 18.52; 95% CI, 9.15-27.89; *P* = .001) and WAT groups (MD, 21.19; 95% CI, 7.79 to 34.60; *P* = .003), but not for the control group (MD, 9.25; 95% CI, –0.84 to 19.34; *P* = .06) (Table 3).

**Table 2.** Changes in Primary and Secondary Outcome Measures at Postintervention or 90 Days Between Groups

Outcome by measurement time	Participant group, median score (IQR)			WA vs control		WAT vs WA	
	Control	WA	WAT	MD (95% CI)	*P* value	MD (95% CI)	*P* value
Life Space Assessment^a^							
Baseline	18.0 (8.0 to 32.5)	22.0 (9.0 to 43.0)	23.5 (10.0 to 34.0)	NA	NA	NA	NA
90 d	29.0 (22.0 to 43.0)	49.5 (37.5 to 59.0)	48.2 (35.5 to 62.0)	12.77 (1.06 to 24.54)	.03	1.52 (−9.59 to 12.83)	.78
1-Minute sit-to-stand test^b^							
Baseline	2.0 (1.0 to 5.0)	2.0 (1.0 to 3.2)	2.0 (1.0 to 3.0)	NA	NA	NA	NA
90 d	12.0 (10.0 to 24.2)	19.0 (9.0 to 21.5)	19.0 (13.5 to 25.0)	8.45 (4.34 to 12.56)	.001	3.36 (−4.97 to 11.71)	.41
Katz Index^c^							
Baseline	1.0 (0 to 3.0)	1.5 (0 to 3.0)	1.0 (1.0 to 2.0)	NA	NA	NA	NA
90 d	1.0 (0 to 1.0)	0 (0 to 0.5)	0 (0 to 1.2)	−0.43 (−1.13 to 0.26)	.20	0.18 (−0.31 to 0.68)	.45
Barthel Index^d^							
Baseline	100 (90.0 to 100)	92.5 (85.0 to 100)	90.0 (80.0 to 100)	NA	NA	NA	NA
90 d	95.0 (85.0 to 100)	100 (100 to 100)	95.0 (85.0 to 100)	8.93 (2.54 to 15.33)	.007	7.92 (1.43 to 14.42)	.02
Lawton-Brody Scale^e^							
Baseline	24.0 (20.2 to 26.0)	23.0 (16.0 to 25.0)	23.0 (20.2 to 26.0)	NA	NA	NA	NA
90 d	25.0 (16.0 to 26.0)	26.0 (23.5 to 26.0)	25.0 (21.0 to 27.0)	1.67 (−1.00 to 4.34)	.21	0.52 (−2.01 to 3.05)	.68
Timed Up and Go test^f^							
Baseline	15.0 (12.7 to 19.2)	16.0 (11.7 to 25.5)	18.0 (14.2 to 25.5)	NA	NA	NA	NA
Postintervention	16.0 (12.2 to 19.5)	10.0 (9.0 to 15.0)	12.0 (10.0 to 16.0)	−10.45 (−22.70 to −1.80)	.02	−0.20 (−12.97 to 12.57)	.97
Falls Efficacy Scale International^g^							
Baseline	22.5 (19.0 to 30.0)	24.0 (21.0 to 30.0)	27.0 (24.0- 33.0)	NA	NA	NA	NA
Postintervention	22.5 (19.0 to 30.0)	22.5 (20.0 to 30.0)	25.0 (22.0 to 30.0)	NA	NA	NA	NA
90 d	25.0 (17.5 to 31.5)	19.0 (16.2 to 21.0)	19.0 (16.0 to 23.0)	−5.60 (−9.06 to −2.14)	.002	−2.29 (−5.90 to 1.32)	.20
Euro Quality of Life Instrument, 5-Dimensions, 3-Level^h^							
Baseline	80.0 (70.0 to 80.0)	72.5 (70.0 to 80.0)	70.0 (65.0 to 80.0)	NA	NA	NA	NA
90 d	77.5 (70.0 to 80.0)	80.0 (70.0 to 92.2)	80.0 (70.0 to 80.0)	−1.17 (−7.34 to 4.99)	.70	5.24 (−0.82 to 11.31)	.08
10-point Cognitive Screener^i^							
Baseline	6.0 (4.0 to 8.2)	6.0 (3.0 to 7.0)	6.0 (4.0 to 8.0)	NA	NA	NA	NA
90 d	6.0 (4.0 to 7.5)	8.0 (7.0 to 9.0)	7.5 (6.5 to 9.0)	1.74 (0.16 to 3.32)	.03	0.02 (−1.52 to 1.57)	.97
15-item Geriatric Depression Scale^j^							
Baseline	2.0 (1.0 to 4.0)	3.0 (1.0 to 4.0)	3.0 (1.0 to 4.0)	NA	NA	NA	NA
90 d	2.0 (1.0 to 3.0)	2.0 (1.0 to 3.2)	2.0 (1.0 to 3.5)	0.02 (−1.65 to 1.61)	.98	0.40 (−1.19 to 2.00)	.61

Abbreviations: MD, mean difference; NA, not applicable; WA, walking aid; WAT, walking aid with telemonitoring.

^a^ Scores range from 0 (bedroom-bound) to 120 (frequent independent travel beyond the city).

^b^ Measured as the number of times participants stand up and sit down completely in 1 minute.

^c^ Scores range from 0 (independence) to 6 (total dependence).

^d^ Scores range from 0 to 100, with higher scores (80-100) indicating greater independence.

^e^ Scores ranging from 9 (totally dependent) to 27 (independent).

^f^ Scored as the time to stand up from the chair, walk 3 m, go around a cone, and then return to the chair and sit down.

^g^ Total scores range from 16 to 64, with higher scores indicating worse levels of efficacy and control.

^h^ Scores range from 0 (the worst health you can imagine) to 100 (the best health you can imagine).

^i^ Final scores, adjusted according to educational level, can indicate normal cognition (≥8 points), possible cognitive impairment (6-7 points), or probable cognitive impairment (0-5 points).

^j^ A total score higher than 5 indicates clinically significant symptoms; 6 to 10, mild depression; and 11 to 15, severe depression.

**Table 3.** Change in Primary and Secondary Outcome Measures From Baseline to Postintervention or 90 Days in Within-Group Analyses

Outcome	Change within group					
	Control		WA		WAT	
	MD (95% CI)	*P* value	MD (95% CI)	*P* value	MD (95% CI)	*P* value
**90-d vs Baseline**						
Life Space Assessment^a^	9.25 (−0.84 to 19.34)	.06	18.52 (9.15 to 27.89)	.001	21.19 (7.79 to 34.60)	.003
1-Minute sit-to-stand test^b^	10.36 (4.92 to 15.80)	.002	12.59 (8.97 to 16.93)	.001	16.52 (12.85 to 20.19)	.001
Katz Index^c^	−0.35 (−0.85 to 0.15)	.16	−0.95 (−1.52 to −0.37)	.002	−0.83 (−1.30 to −0.36)	.003
Barthel Index^d^	−3.33 (−6.40 to −0.25)	.03	9.73 (1.92 to 17.54)	.02	6.39 (2.99 to 9.82)	.002
Lawton-Brody Scale^e^	1.06 (−2.84 to 4.96)	.57	1.55 (0.08 to 3.02)	.03	1.46 (0.07 to 2.85)	.008
Falls Efficacy Scale International^f^	0.65 (−1.98 to 3.28)	.61	−4.84 (−6.12 to −3.56)	.001	−8.05 (−11.11 to −4.98)	<.001
Euro Quality of Life Instrument, 5-Dimensions, 3-Level^g^	−3.21 (−9.68 to 3.25)	.30	10.66 (4.13 to 17.20)	.002	11.06 (5.83 to 16.30)	<.001
10-point Cognitive Screener^h^	−0.33 (−2.08 to 1.42)	.70	1.76 (0.84 to 2.68)	.001	2.31 (0.59 to 4.02)	.008
15-item Geriatric Depression Scale^i^	−0.33 (−1.83 to 1.16)	.65	−0.94 (−1.24 to −0.65)	.001	−0.95 (−1.30 to −0.60)	<.001
**Postintervention vs baseline**						
Timed Up and Go test^j^	6.47 (−6.72 to 19.67)	.31	−5.26 (−9.16 to −1.36)	.01	−7.06 (−12.64 to −1.47)	.01

Abbreviations: WA, walking aid; WAT, walking aid with telemonitoring.

^a^ Scores range from 0 (bedroom-bound) to 120 (frequent independent travel beyond the city).

^b^ Measured as the number of times participants stand up and sit down completely in 1 minute.

^c^ Scores range from 0 (independence) to 6 (total dependence).

^d^ Scores range from 0 to 100, with higher scores (80-100) indicating greater independence.

^e^ Scores ranging from 9 (totally dependent) to 27 (independent).

^f^ Total scores range from 16 to 64, with higher scores indicating worse levels of efficacy and control.

^g^ Scores range from 0 (the worst health you can imagine) to 100 (the best health you can imagine).

^h^ Final scores, adjusted according to educational level, can indicate normal cognition (≥8 points), possible cognitive impairment (6-7 points), or probable cognitive impairment (0-5 points).

^i^ A total score higher than 5 indicates clinically significant symptoms; 6 to 10, mild depression; and 11 to 15, severe depression.

^j^ Scored as the time to stand up from the chair, walk 3 m, go around a cone, and then return to the chair and sit down.

#### Fear of Falling

At 90 days, the WA group demonstrated a significant reduction in FES-I scores compared with the control group (MD, −5.60; 95% CI, −9.06 to −2.14; *P* = .002), with no significant difference compared with the WAT group (MD, −2.29; 95% CI, −5.90 to 1.32; *P* = .20) (Table 2). Within-group analyses showed significant reductions from baseline to 90 days in both the WA group (MD, −4.84; 95% CI, −6.12 to −3.56; *P* = .001) and the WAT group (MD, −8.05; 95% CI, −11.11 to −4.98; *P* < .001), whereas no significant change was observed in the control group (MD, 0.65; 95% CI, −1.98 to 3.28; *P* = .61) (Table 3).

### Secondary Outcomes

#### Functional Capacity

At 90 days, the WA group had a significant increase in repetitions on the 1-minute sit-to-stand test compared with the control group (MD, 8.45; 95% CI, 4.34-12.56; *P* = .001), but there was no significant difference when compared with the WAT group (MD, 3.36; 95% CI, −4.97 to 11.71; *P* = .41) (Table 2). Within-group analyses revealed significant improvements from baseline to 90 days in the WA group (MD, 12.59; 95% CI, 8.97-16.93; *P* = .001), WAT group (MD, 16.52; 95% CI, 12.85-20.19; *P* = .001), and control group (MD, 10.36; 95% CI, 4.92-15.80; *P* = .002) (Table 3).

For Katz Index scores, no significant differences were observed at 90 days between the WA and control groups (MD, −0.43; 95% CI, −1.13 to 0.26; *P* = .20) or between the WAT and WA groups (MD, 0.18; 95% CI, −0.31 to 0.68; *P* = .45) (Table 2). However, within-group analyses showed significant improvements from baseline to 90 days in the WA group (MD, −0.95; 95% CI, −1.52 to −0.37; *P* = .002) and WAT group (MD, −0.83; 95% CI, −1.30 to −0.36; *P* = .003), but not in the control group (MD, −0.35; 95% CI, −0.85 to 0.15; *P* = .16) (Table 3).

#### Other Secondary Outcomes

Secondary outcomes for other functional capacity measures (Barthel Index and Lawton and Brody Scale), gait assessment (TUG test), quality of life (EQ-5D-3L), cognition (10-CS), depression (GDS-15), and occurrence of falls are presented in eTables 1 and 2 and the eResults in Supplement 2.

## Discussion

This randomized clinical trial addressed the importance of targeted interventions to improve mobility and quality of life in older patients after discharge from the ED. We found that a program of training and the provision of walking aids resulted in significant improvements in mobility, gait, functional capacity, fear of falling, and cognition. However, telemonitoring did not provide additional benefit.

One of the key findings of our study is the improvement in mobility in living spaces in the groups receiving training in walking aids This choice of primary outcome is innovative, particularly in the context of the ED, where the focus is usually on the occurrence of falls and associated injuries.^52,53,54,55^ The LSA has been validated in community-dwelling older adults and is associated with outcomes such as mortality, hospitalizations, and cognitive decline.^56^ Previous studies indicate that reduced mobility precedes limitations in ADL, making it a critical factor for elderly functionality.^56^ In our study, an increase in LSA scores reflected improved mobility across different living spaces, from the home environment to outdoor settings.^57^ The 90-day improvement in mobility scores in the intervention groups also highlights the impact of a program that includes the prescription, training, and provision of gait-assistive devices by specialized professionals in a geriatric ED. Additionally, both the WA and WAT groups had an improvement greater than 5 points in the LSA score, which exceeds the minimum clinically important difference for the LSA scale.^52^ In contrast to a Canadian study associating walking aid use with functional decline in older patients with minor injuries,^58^ this trial demonstrated improvements in the Katz Index, Barthel Index, and Lawton-Brody Scale scores in the WA and WAT groups, reinforcing the effectiveness of the interventions on functional capacity.

Furthermore, studies have confirmed that mobility is directly related to quality of life,^59^ and gait speed, balance, and strength are among the physical measures most strongly associated with quality of life.^60^ Fear of falling significantly affects mobility, potentially leading to activity restriction, gait limitation, depression, and impairment in ADL.^61,62^ In the present study, a reduction in FES-I scores was observed alongside improvements in gait, life-space mobility, and quality of life. However, despite the consistency with previous findings, no formal association analyses were conducted to establish statistical relationships between these variables.

The concomitant improvement in mobility, functional capacity, depression, and cognition reinforces the relevance of comprehensive interventions for the health of acutely ill older adults. Our findings align with studies that demonstrate the relationship among social activity, decreased risk of disability in ADLs, and improved mobility.^57,63^ To our knowledge, this randomized clinical trial is the first to demonstrate the effect of training and providing walking aids, with or without telemonitoring, in a geriatric ED, aligning with international guidelines aimed at promoting autonomy and quality of life in older adults.

### Limitations

Among the study’s limitations, the single-center design may limit its generalizability. However, its detailed description and registration on the ClinicalTrials.gov platform may enable future replications in different populations and settings. Another limitation is the inability to blind participants due to the obvious nature of the intervention. However, blinding of the statistician and the researcher responsible for outcome assessment minimized the risk of bias.

## Conclusions

In this randomized clinical trial, provision of walking aids with training by a physiotherapist in the ED improved mobility, fear of falling, gait, functionality, and cognition in older adults at 90 days. While telemonitoring did not demonstrate incremental benefit, these findings support the integration of specialized geriatric assessment and intervention in the ED to optimize functional recovery following discharge.

## Notes

Supplement 1.
Trial Protocol

Supplement 2. **eMethods 1**. Guidance for Safe Gait **eMethods 2.** Criteria for Determining the Most Appropriate Walking Aid **eResults.** Secondary Outcomes: Functional Capacity, Gait Assessment, Quality of Life, Cognition, Depression, and Occurrence of Falls **eTable 1.** Quality of Life According to Levels and Dimensions of EQ-5D-3L **eTable 2.** Occurrence of Falls at 90 Days

Supplement 3.
Data Sharing Statement
