# Supplementary material for: Walking Aids and Locomotion Training in the Emergency Department: A Randomized Clinical Trial
Source: JAMA Netw Open. 2025 Nov 21;8(11):e2544535. doi: 10.1001/jamanetworkopen.2025.44535 (PMC12639483; doi:10.1001/jamanetworkopen.2025.44535)
Supplement: Supplement 1. — Trial Protocol [file jamanetwopen-e2544535-s001.pdf]

TRAINING AND PROVISION OF MOBILITY AIDS TO PROMOTE AUTONOMY AND MOBILITY OF OLDER PATIENTS IN A GERIATRIC EMERGENCY DEPARTMENT: A PROTOCOL FOR A RANDOMIZED CONTROLLED TRIAL

Research Team:

Pesquisadores responsáveis:

Fernanda Sato Polesel
Email: fernanda.polesel@hsl.org.br

Sâmia Denadai
Email: samia.denadai@hsl.org.br

Marlon Juliano Romero Aliberti
Email: mjaliberti@slserv.com.br

Christian Valle Morinaga
Email: christian.morinaga@hsl.org.br

Mario Chueire de Andrade-Junior
Email: mariochueire@gmail.com

Itiana Cardoso Madalena
Email: itiana.madalena@hsl.org.br

Wellington Pereira Yamaguti
Email: wellington.psyamaguti@hsl.org.br

Pedro Kallas Curiati
Email: pedro.kcuriati@hsl.org.br

Renato Fraga Righetti
Email: renato.righetti@hsl.org.br

ABSTRACT

INTRODUCTION: Older adults have higher rates of emergency department (ED) admissions when compared to their younger counterparts. Mobility is the ability to move around, but also encompasses the environment and the ability to adapt to it. Walking aids can be used to improve mobility and prevent falls. According to international guidelines, they must be available in Geriatric EDs. This study aims to evaluate the efficacy of a program of training and provision of walking aids (WA), associated or not with telemonitoring, on fear of falling, mobility, quality of life and risk of falls up to 3 months in older adults cared for in an ED. **METHODS:** A randomized clinical trial will be carried out in the ED. Participants will be randomized and allocated into three groups, as follows: A) walking aid group; B) walking aid and telemonitoring group; C) Control group. Patients will undergo a baseline evaluation encompassing sociodemographic and clinical data, mobility in life spaces (Life Space Assessment), gait speed, muscle strength, functionality (Barthel Index, Katz index, and Lawton Scale), quality of life (Euro Quality of Life Instrument-5D), fear of falling (Falls Efficacy Scale International), history of falls, cognition (10-Point Cognitive Screener) and mood (15-point Geriatric Depression Scale) before the intervention. Gait time and fear of falling will be assessed again after the intervention. Finally, mobility in life spaces, functionality, quality of life, fear of falling, history of falls, cognition, and mood will be assessed 3 months after discharge from the geriatric ED through a telephone interview. For statistical analysis, paired T-test, Wilcoxon test, One-Way ANOVA, Kruskall-Wallis, Pearson correlation, and Spearman test will be used according to the normality established by the Shapiro-Wilk normality test. Results will be considered significant when $P<0.05$.

Keywords: clinical trial, geriatric, mobility, life space, walking aids, emergency room

INTRODUCTION

According to American Geriatric emergency department (ED) Guidelines, proper care for older patients requires policies, protocols, and flows designed for the specific needs of this population [1]. The European geriatric ED guidelines quote as main recommendations for this population the “5 Ms of Geriatrics”: mind, medication, multi-complexity, “most important” and mobility. In other words, during care for older adults in ED, it is recommended: approach to dementia, delirium, depression and cognitive impairment; structured assessment and review of medications used by older adults, considering possible drug interactions and inappropriate use of medication; consideration of general needs of the older adults, as medical, psychological, social, functional or environmental; regarding item “most important” it is recommended ensure the heath results individually focusing on objectives that are meaningful to the older adults [2]. It is also recommended that geriatric ED physical space focus on structural modifications aimed at safety, comfort, memory cues and sensorial perception (both vision and hearing), ensuring greater mobility for older adults. Furthermore, guidelines emphasize that mobility and safety improvement, are not only related to furniture, but also reinforce the importance of easy access to walking aids [1].

Mobility limitations are common in older adults and are associated with depressive symptoms and decrease quality of life, culminate in decrease social interaction, isolation and loneliness, affecting physical, psychological and social aspects of older adults [3]. Risk factors that are more associated with mobility limitation are advanced age, low physical activity, obesity, strength and balance impairment, gait alteration and chronic diseases, in addition to other factors less reported such as depressive symptoms and cognitive impairment, using alcohol or tobacco, recent hospitalization [4]. Therefore, mobility assessment should be considered as a component to be included in older adults heath care [4].

Interventions with walking aids promote independency through biomechanical stabilization, balance and control motor improvement, sensorial feedback, decrease load on the lower limbs, decrease fear falling and fall prevention [5, 6]. Furthermore, several studies point telemonitoring as a tool for health care [7]. It is defined as the use of information technology and telecommunications for remote health care [8]. In a systematic review published in 2019, it was considered viable and well received for older adults heath care and recommended for clinical practice because overcome barriers of distance and access to health services [9]. Few studies have explored telemonitoring for population in ED service, despite evidence that this intervention with older adults with multiple diseases reduce the number of hospitalizations and ED visit [10, 11]. In fact, there is no data on the effects of an intervention encompassing gait devices, associated or not with telemonitoring, to improve the mobility of older patients admitted to the ED.

The hypothesis of the study is that the provision of walking aids enhances mobility in living spaces and will have a significant impact on fear of falling, functionality, and quality of life. Therefore, this study aims to evaluate the efficacy of a program of training and provision of walking aids, associated or not with telemonitoring, on fear falling, mobility, quality of life, and risk of falls up to 3 months in older adults cared for in an ED.

Objective

Primary objective

Evaluate the efficacy of a program of training and provision of walking aids, associated or not with telemonitoring, on fear falling, mobility and balance in older adults cared for in an ED.

Secondary objective

Evaluate the efficacy of a program of training and provision of walking aids, associated or not with telemonitoring, on quality of life and risk of falls up to 3 months in older adults cared for in an ED.

METHODS

Study Design

Initially, the amendment to the research project will be submitted to the Research Ethics Committee of Hospital Sírio-Libanês. The protocol of this research project will be registered on "ClinicalTrials.gov" platform as a way of providing information regarding this clinical trial, as well as ensuring transparency in the conduct of stages of this study. This will be a randomized and blind clinical trial. It will be conducted on facilities of Hospital Sírio-Libanês (HSL) – Bela Vista Unit, an institution located in the city of São Paulo, Brazil.

Population

Older adults who will be receiving care in ED of HSL will be recruited.

Sample Size Calculation

Sample size calculation was based on the study by Kennedy et al. (2018), which reported a minimum clinically important difference of 5 points on the LSA scale, with a standard deviation of 5.1 points.The total sample size was determined to be 66 individuals for an α of 0.05 and a power of 0.80. Estimating a 15% loss to follow-up, the sample size needed for the study's success was increased to 75 individuals, randomized (1:1:1) into 25 participants in each group.

Inclusion Criteria

For patients who sign Informed Consent Term, following inclusion criteria will be adopted: aged 65 years or older, admitted to the Geriatric ED of HSL, with at least one criteria of institutional protocol for indication and training of mobility aids: reduction of postural instability; improvement of motor control; increase of somatosensory feedback; reduction of biomechanical overload; safe promotion of autonomy; fall history (in the last three months).

Exclusion Criteria

Exclusion criteria will be: altered level of conscience, need for supplemental oxygen (≥3L/min), respiratory distress, hemodynamic instability, postural instability with a tendency to fall backward, cognitive impairment that limits the use of walking aids, hospitalization after ED evaluation, and delirium.

Randomization and allocation

The process of randomization will be carried out by the software *"Research Electronic Data Capture"* (REDCap), ensuring a similar chance to be allocate to any of intervention groups (1:1:1). Randomization will determine allocation in three groups: walking aid (WA) group; walking aid with telemonitoring (WAT) group; control group.

- Groups

- **Walking aid (WA) group:** patients will be trained for the use of a walking aid and receive guidance on safe gait but will not receive telemonitoring.
- **Walking aid with telemonitoring (WAT) group:** patients will receive training for the use of a walking aid, guidance on safe gait, and telemonitoring.
- **Control group:** patients will receive only guidance on safe gait and will not receive training for the use of walking aid or telemonitoring.

- Blinding

This study will be blind for the researchers involved in the assessments at 3 months and the researcher who will analyze the data.

Procedures

- Recruitment

Participants will be recruited in the Geriatric ED of HSL, Bela Vista Unit, São Paulo, Brazil, by a trained research assistant, who will remain on duty for 25 hours per week in shifts distributed between 7:00 and 18:00 for up to 3 months. This may be triggered by physician or nurse on duty, but researcher will also actively search for potential candidates for the study. Patients eligible for screening for the study will be contacted by researcher and will signed the Informed Consent Term.

- Screening

Initially, all volunteer participants will undergo a screening process to ensure the eligibility criteria for this study. In this step, the participants are going to be interviewed for data collection (social, demographic, clinical, and medication history). Also, delirium will be considered as an exclusion criteria [13, 14]. For this evaluation will be used Confusion Assessment Method (CAM) [15].

- Assessment Moments

Baseline assessment will be carried out before interventions. Assessment of gait speed and fear of falling will be repeated immediately after the intervention. Fear of falling, life-space mobility, functionality, quality of life, fall history and cognition

assessments will be repeated 3 months after intervention by a telephone interview or video call.

- Fear of Falling

Fear of falling will be evaluated by Falls Efficacy Scale International (FES-I) [16]. This tool has 16 items that evaluate, for example, walking on a slippery surface or an uneven surface, visiting a friend/relative, or attending a social event [17]. The fear of falling in each activity is classified on a 4-point scale (1 - not at all concerned to 4 - very concerned) [17]. The total score ranges from 16 to 64: 16-22 (low concern), 20-27 (moderate concern), and 28-64 (high concern) [17].

- Mobility in life-space

Mobility in life space will be assessed by the Life Space Assessment (LSA), which allows the characterization of mobility in life-spaces (other rooms of your home besides the room where you sleep; to an area outside your home, places outside your neighborhood but within your town; and to places outside your town), specifically frequency, need for mobility aids and the help of third party in the last 4 weeks [18]. The score reflects the distance, frequency and level of independence, with a range from 0 to 120 [19].

- Gait Assessment

Timed Up and Go test (TUG) evaluate mobility, balance, gait, and risk of falling [20]. In TUG, the researcher requests the participant to stand up on the chair (without using arms), walk 3 meters, go around outside a cone, walk back to the chair, and sit down without using arms [21].

- Peripheral Muscle Strength

Peripheral muscle strength will be measured by handgrip strength (HGS) with a hand-held dynamometer (model SH 5001, brand SAEHAN) [22], respecting the protocol recommended by the American Association of Hand Therapists (ASHT) [23]. The best score between the three measures is going to be considered a handgrip strength measure. For this assessment will be used the dynamometer from the HSL physical therapy research team.

- Functional capacity

One-minute sit-to-stand test will be based on a protocol described by Ozalevli and cols [24]. With the hands on the hip, participants will be guided to sit and stand completely in a chair (height 46 centimeters) as often as possible during 1 minute [24]. To basic activities of daily living (ADL) assessment will be used the Katz Index [25]. As a result, participants will be classified from 0 (independency for ADL) to 6 (total dependency for ADL) [26, 27]. The Barthel index evaluates the autonomy for self-care, in addition to mobility [28-30]. The final score ranges from 0 to 100 and each item is scored according to how the individual performs each task (independently, with some help, or dependently) [28-30]. The higher the score, the higher the independency: 80 to 100 indicate independence; 60-79 points for slight dependency; 40-59 points for moderate independence; 20-39 points for severe dependency; 0-20 points for total dependency [28-30]. Instrumental ADL will be evaluated by the Lawton-Brody scale, which includes seven activities: the ability to use the telephone, mode of transportation, shopping, housekeeping, food preparation, responsibility for own medications, and ability to handle finances [31]. The final score classifies the individuals as independent (25 to 27 points), mild dependent (21 to 25 points), moderate dependent (16 to 20 points), severe dependent (10 to 15 points) and totally dependent (9 points) [31].

- Quality of Life

Quality of life will be evaluated by Euro Quality of Life Instrument-5D (EQ-5D) [32]. This instrument covers five health dimensions: mobility, self-care, usual activities, pain/discomfort, and anxiety/depression [33]. Each dimension is classified into 3 levels: no problems (1 point), some problems (2 points), and extreme problems (3 points) [33]. In the end, the instrument has self-rated health on a visual analog scale ranging from 0 (worst imaginable health state) to 100 (best imaginable health state), resulting in 243/3125 health state distinct [32].

- Fall History

Fall history will be evaluated periodically during the study and the patients must fill out a diary to register every moment in which they fall [34]. For each fall, location, associated injuries, need for special care after the fall will be evaluated.

- Cognition and Mood Assessment

Cognitive assessment will be performed using the 10-Point Cognitive Screener (10-CS) [35]. This tool covers an evaluation of temporal orientation (year, month and day), verbal fluency (naming animals in 1 minute), and three-word recall (as glasses, pen and hammer) learned before the distraction maneuver [35]. Results are interpreted as follows: ≥ 8 points – normality, 6 to 7 points - possible cognitive impairment (usually mild cognitive impairment), and 0 to 5 points - probable cognitive impairment (usually dementia) [35]. Assessment of mood disorders will be evaluated by the Geriatric Depression Scale (GDS-15) [36], which consists of 15 items (possible answers Yes or No) and a score of 0 or 1 [36]. The final score greater or equal to 5 points indicates the presence of significant symptoms of depression [36].

- Geriatric Vulnerability and Frailty Assessment

Geriatric vulnerability will be evaluated by the PRO-AGE scoring system, a fast tool developed to assess older patients in the ED [38]. For the predictive model for hospital admission will be collected variables of presence of functional decline (4 points), recent hospitalization (2 points), advanced age (1 point), acute mental change (3 points), weight loss (2 points) and male patient (1 point). For the predictive model for prolonged hospital stay and in-hospital death will be collected variables of functional decline (2 points), recent hospitalization (1 point), advanced age (1 point), weight loss (1 point) and fatigue (1 point) [38]. Frailty will be evaluated using the Clinical Frailty Scale (CFS). Patients will be classified in one of the following categories: 1- very fit; 2- well; 3- managing well; 4- vulnerable; 5- mildly frail; 6- moderately frail; 7- severely frail; 8- very severely frail; 9- terminally ill [39].

Figure 1. Flow diagram of the clinical trial

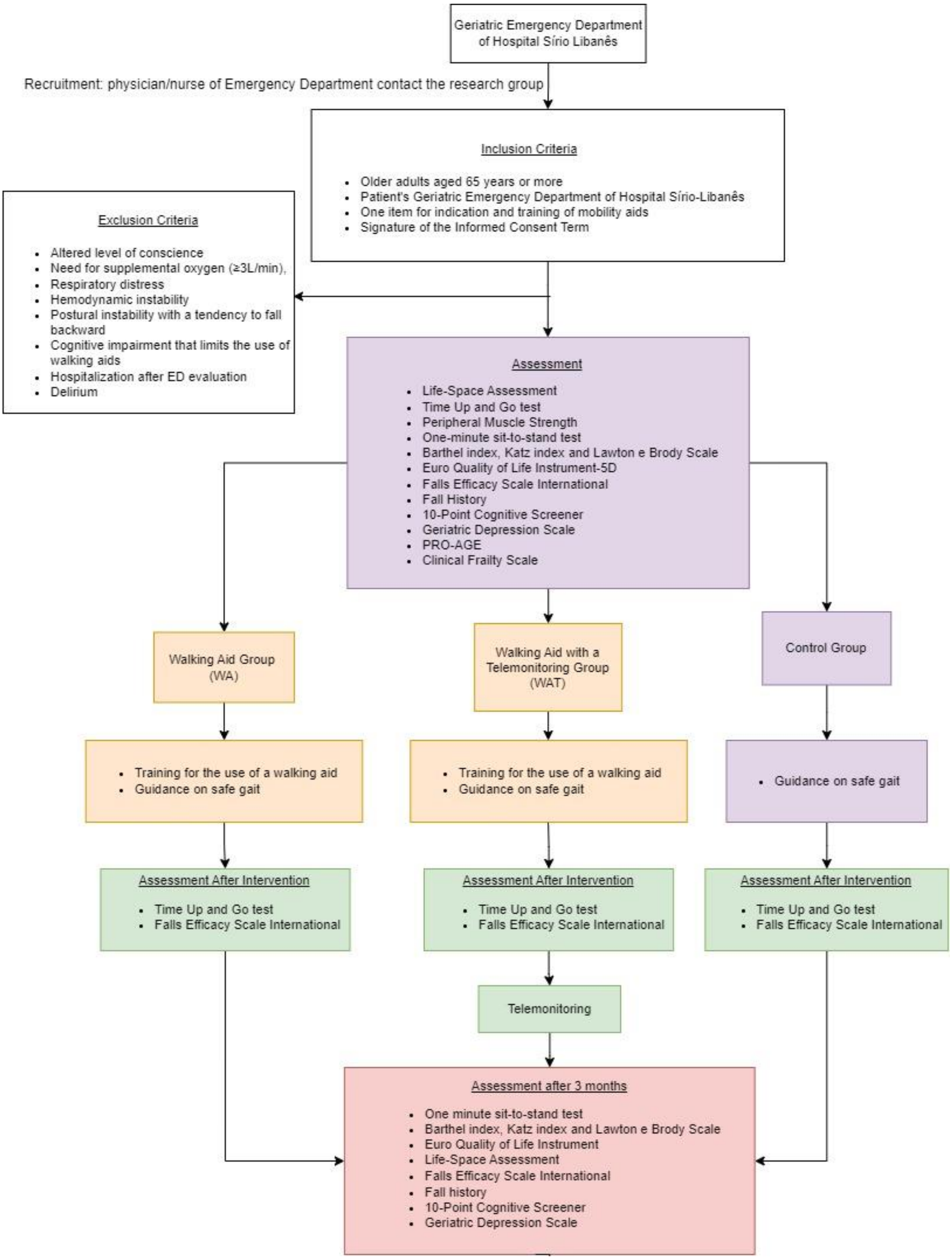

Interventions

The interventions will be performed according to the randomization after the baseline data collection.

1. Training of walking aids

This training will be carried out with patients from groups WA and WAT. After data collection, physical therapist will identify the mobility needs and will indicate the most appropriate walking aid. It will be considered the following needs related for each aid: (A) Canes: one upper limb used for a walk, light weight-bearing and need for somatosensorial feedback; the cane must be positioned between 15 and 20 centimeters laterally to the feet; the patient's hand should be supported on the cane at the height of the greater trochanter of the femur and the elbow should be flexed approximately by 30º; in general, the cane is used on the opposite side of the injured leg. (B) Walkers: both upper limbs used to walk, heavy weight bearing, and presence of postural instability; the equipment must be held between 20 and 25 centimeters in front of the body with relaxed shoulders, erect torso, and elbow flexed at 20º to 30º;

2. *Telemonitoring*

*This f*ollow-up will be carried out only in the WAT group. Telemonitoring will occur every two weeks for three months after the ED discharge, through video call (about 15 minutes), the importance of using mobile devices and the guidance on safe gait will be reinforced. If unavailability on first contact, two more attempts will be made on subsequent days.

3. Guidance on Safe Gait

Guidance on safe gait will be carried out for all the groups in the study. Subjects will be instructed on strategies for fall prevention [40] and will receive a printed leaflet summarizing for getting up, walking and climbing stairs.

Statistical Analysis

Continuous variables will be expressed on average and standard deviation or median and interquartile interval 25%-75%. Categorical data will be presented in

absolute and relative numbers. To verify the distribution normality of data, the Shapiro-Wilk normality test will be applied. Paired t-test for parametric data or a Wilcoxon test for non-parametric data will be used. Evaluation between the three intervention groups the One-way ANOVA test for parametric data or Kruskall-Wallis for non-parametric data will be used. To evaluate correlations the Pearson correlation test will be used for parametric data and the Spearman test will be used for non-parametric data. All the analyzes will be carried out using the Statistical Package for Social Sciences (SPSS) version 28.0.1 (SPSS Inc.®; Chicago, IL, USA), considering a significance level of 5%.

Ethics approval and consent to participate

This study received ethical approval from the Research Ethics Committee of the Hospital Sírio-Libanês. The study will be conducted in accordance with national and international resolutions as described in Resolution nº 466 of December 12, 2012 and in the Declaration of Helsinki and all its revisions and amendments. Informed Consent Form will be applied directly to the voluntary participant, certifying agreement to participate and be included in the study.

Potential benefits and risks of carrying out the study

Potential benefits of this study will be identification of older adults with gait changes and implementation of an intervention with the aim of increasing the mobility of older adults and minimizing the risk of falling. Regarding risks, volunteer participants in this study may report different degrees of discomfort, tiredness or fatigue, especially during performance of functional and strength tests. However, these symptoms should cease after 5 minutes of rest. All information collected will be compiled and managed through the electronic data capture system (REDCap) hosted on the servers of Hospital Sírio-Libanês.

Expected Results

Fear of falls among older adults can result in a significant reduction in their mobility. This apprehension can lead older adults to avoid activities and spaces that they consider risky, thus limiting their social participation and their ability to move independently. However, an effective intervention in the ED of these older adult involving the provision and training of walking aids can help reduce their fear of falling and improve their mobility in the environment. Furthermore, the use of telemonitoring can enhance these results, allowing remote and continuous monitoring of the health and well-being of older adults, offering additional support and contributing to greater security and confidence in carrying out their daily activities.

Funding

The study was funded by the Geriatric Center for Advanced Medicine, Hospital Sírio-Libanês, São Paulo.

References

1. American College of Emergency Physicians; American Geriatrics Society; Emergency Nurses Association; Society for Academic Emergency Medicine; Geriatric Emergency Department Guidelines Task Force. Geriatric emergency department guidelines. Ann Emerg Med. 2014;63(5):e7-25.

2. Lucke JA, Mooijaart SP, Heeren P, Singler K, McNamara R, Gilbert T, et al. Providing care for older adults in the Emergency Department: expert clinical recommendations from the European Task Force on Geriatric Emergency Medicine. Eur Geriatr Med. 2022;13(2):309-317.

3. James BD, Boyle PA, Buchman AS, Bennett DA. Relation of late-life social activity with incident disability among community-dwelling older adults. J Gerontol A Biol Sci Med Sci. 2011 Apr;66(4):467-73.

4. Brown CJ, Flood KL. Mobility limitation in the older patient: a clinical review. JAMA. 2013;310(11):1168-77.

5. Bateni H, Maki BE. Assistive devices for balance and mobility: benefits, demands, and adverse consequences. Arch Phys Med Rehabil. 2005 Jan;86(1):134-45.

6. Omana H, Madou E, Divine A, Wittich W, Hill KD, Johnson AM, Holmes JD, Hunter SW. The Differential Effect of First-Time Single-Point Cane Use between Healthy Young and Older Adults. PM R. 2021 Dec;13(12):1399-1409.

7. van den Berg N, Schumann M, Kraft K, Hoffmann W. Telemedicine and telecare for older patients--a systematic review. Maturitas. 2012 Oct;73(2):94-114.

8. Merrell RC. Geriatric Telemedicine: Background and Evidence for Telemedicine as a Way to Address the Challenges of Geriatrics. Healthc Inform Res. 2015;21(4):223-9.

9. Batsis JA, DiMilia PR, Seo LM, Fortuna KL, Kennedy MA, Blunt HB, Bagley PJ, Brooks J, Brooks E, Kim SY, Masutani RK, Bruce ML, Bartels SJ. Effectiveness of Ambulatory Telemedicine Care in Older Adults: A Systematic Review. J Am Geriatr Soc. 2019 Aug;67(8):1737-1749.

10. Takahashi PY, Pecina JL, Upatising B, Chaudhry R, Shah ND, Van Houten H, Cha S, Croghan I, Naessens JM, Hanson GJ. A randomized controlled trial of telemonitoring in older adults with multiple health issues to prevent hospitalizations and emergency department visits. Arch Intern Med. 2012 May 28;172(10):773-9.

11. Gellis ZD, Kenaley BL, Ten Have T. Integrated telehealth care for chronic illness and depression in geriatric home care patients: the Integrated Telehealth Education and Activation of Mood (I-TEAM) study. J Am Geriatr Soc. 2014 May;62(5):889-95.

12. Kennedy R, Almutairi M, Williams C, Sawyer P, Allman R, Brown C. WHAT IS THE MINIMUM CLINICALLY IMPORTANT DIFFERENCE FOR LIFE-SPACE? *Innov Aging*. 2018;2(suppl_1):463-463.

13. Kahn JH, Magauran BG Jr, Olshaker JS, Shankar KN. Current Trends in Geriatric Emergency Medicine. Emerg Med Clin North Am. 2016 Aug;34(3):435-52.

14. Organization WH. International Statistical Classification of Diseases and Related Health Problems 10th Revision (ICD-10). F05 Delirium, not induced by alcohol and other psychoactive substances. http://apps.who.int/classifications/icd10/browse/2016/en2016.

15. 13. Inouye SK. Delirium-A Framework to Improve Acute Care for Older Persons. J Am Geriatr Soc. 2018;66(3):446-51.

16. Tinetti ME, Richman D, Powell L. Falls efficacy as a measure of fear of falling. J Gerontol. 1990;45(6):P239-43.

17. França AB, Low G, de Souza Santos G, da Costa Serafim R, Vitorino LM. Psychometric properties of the falls efficacy scale-international and validating the short version among older Brazilians. Geriatr Nurs. 2021;42(2):344-50.

18. Baker PS, Bodner EV, Brown CJ, Kennedy RE, Allman RM. Life-Space Assessment composite score rationale. Clin Rehabil. 2016;30(1):95-7.

19. Simões MDSM, Garcia IF, Costa LDC, Lunardi AC. Life-Space Assessment questionnaire: Novel measurement properties for Brazilian community-dwelling older adults. Geriatr Gerontol Int. 2018 May;18(5):783-789.

20. Barry E, Galvin R, Keogh C, Horgan F, Fahey T. Is the Timed Up and Go test a useful predictor of risk of falls in community dwelling older adults: a systematic review and meta-analysis. BMC Geriatr. 2014;14:14.

21. Bennell K, Dobson F, Hinman R. Measures of physical performance assessments: Self-Paced Walk Test (SPWT), Stair Climb Test (SCT), Six-Minute Walk Test (6MWT), Chair Stand Test (CST), Timed Up & Go (TUG), Sock Test, Lift and Carry Test (LCT), and Car Task. Arthritis Care Res (Hoboken). 2011;63 Suppl 11:S350-70.

22. Rijk JM, Roos PR, Deckx L, van den Akker M, Buntinx F. Prognostic value of handgrip strength in people aged 60 years and older: A systematic review and meta-analysis. Geriatr Gerontol Int. 2016 Jan;16(1):5-20.

23. Desrosiers J, Bravo G, Hébert R, Dutil E. Normative data for grip strength of elderly men and women. Am J Occup Ther. 1995;49(7):637-44.

24. 20. Ozalevli S, Ozden A, Itil O, Akkoclu A. Comparison of the Sit-to-Stand Test with 6 min walk test in patients with chronic obstructive pulmonary disease. Respir Med. 2007;101(2):286-93.

25. Yurkovich M, Avina-Zubieta JA, Thomas J, Gorenchtein M, Lacaille D. A systematic review identifies valid comorbidity indices derived from administrative health data. J Clin Epidemiol. 2015;68(1):3-14.

26. KATZ S, FORD AB, MOSKOWITZ RW, JACKSON BA, JAFFE MW. STUDIES OF ILLNESS IN THE AGED. THE INDEX OF ADL: A STANDARDIZED MEASURE OF BIOLOGICAL AND PSYCHOSOCIAL FUNCTION. JAMA. 1963;185:914-9.

27. Lino VTS, Pereira SRM, Camacho LAB, Ribeiro Filho ST, Buksman S. Adaptação transcultural da Escala de Independência em Atividades da Vida Diária (Escala de Katz). Cad Saúde Pública. 2008;24(1):103–12.

28. Mahoney FI, Barthel DW. Functional evaluation: the Barthel index. Md State Med J. 1965;14: 61-5.

29. Minosso JSM, Amendola F, Alvarenga MRM, Oliveira MA de C. Validação, no Brasil, do Índice de Barthel em idosos atendidos em ambulatórios. Acta paul enferm [Internet]. 2010Mar;23(2):218–23.

30. Loyd C, Markland AD, Zhang Y, Fowler M, Harper S, Wright NC, et al. Prevalence of Hospital-Associated Disability in Older Adults: A Meta-analysis. J Am Med Dir Assoc. 2020;21(4):455-61.e5.

31. Lawton MP, Brody EM. Assessment of older people: self-maintaining and instrumental activities of daily living. Gerontologist. 1969;9(3):179-86.

32. EuroQol Group. EuroQol--a new facility for the measurement of health-related quality of life. Health Policy. 199;16(3):199-208.

33. EQ-5D (Internet). [Accessed: 12 June 2023] Available at: https://euroqol.org/

34. Pasquetti P, Apicella L, Mangone G. Pathogenesis and treatment of falls in elderly. Clin Cases Miner Bone Metab. 2014;11(3):222-5.

35. Apolinario D, Lichtenthaler DG, Magaldi RM, Soares AT, Busse AL, Amaral JR, et al. Using temporal orientation, category fluency, and word recall for detecting cognitive impairment: the 10-point cognitive screener (10-CS). Int J Geriatr Psychiatry. 2016;31(1):4-12.

36. Shin C, Park MH, Lee SH, Ko YH, Kim YK, Han KM, et al. Usefulness of the 15-item geriatric depression scale (GDS-15) for classifying minor and major depressive disorders among community-dwelling elders. J Affect Disord. 2019;259:370-5.

37. Royal College of Physicians. National Early Warning Score (NEWS) 2 [Internet]. Royal College of Physicians. Royal College of Physicians; 2017. Available from: https://www.rcplondon.ac.uk/projects/outputs/national-early-warning-score-news-2.

38. Curiati PK, Gil-Junior LA, Morinaga CV, Ganem F, Curiati JAE, Avelino-Silva TJ. Predicting Hospital Admission and Prolonged Length of Stay in Older Adults in the Emergency Department: The PRO-AGE Scoring System. Ann Emerg Med. 2020;76(3):255-265.

39. Aprahamian I, Cezar NOC, Izbicki R, Lin SM, Paulo DLV, Fattori A, Biella MM, Jacob Filho W, Yassuda MS. Screening for Frailty With the FRAIL Scale: A Comparison With the Phenotype Criteria. J Am Med Dir Assoc. 2017 Jul 1;18(7):592-596.

40. Gillespie LD, Robertson MC, Gillespie WJ, Sherrington C, Gates S, Clemson LM, Lamb SE. Interventions for preventing falls in older people living in the community. Cochrane Database Syst Rev. 2012 Sep 12;2012(9):CD007146.
