# Supplementary material for: Walking Aids and Locomotion Training in the Emergency Department: A Randomized Clinical Trial
Source: JAMA Netw Open. 2025 Nov 21;8(11):e2544535. doi: 10.1001/jamanetworkopen.2025.44535 (PMC12639483; doi:10.1001/jamanetworkopen.2025.44535)
Supplement: Supplement 2. — eMethods 1. Guidance for Safe Gait eMethods 2. Criteria for Determining the Most Appropriate Walking Aid eResults. Secondary Outcomes: Functional Capacity, Gait Assessment, Quality of Life, Cognition, Depression, and Occurrence of Falls eTable 1. Quality of Life According to Levels and Dimensions of EQ-5D-3L eTable 2. Occurrence of Falls at 90 Days [file jamanetwopen-e2544535-s002.pdf]

Supplementary Online Content

Polesel FS, Denadai S, Morinaga CV, et al. Walking Aids and locomotion training in the emergency department: a randomized clinical trial. *JAMA Netw Open.* 2025;8(11):e2544535. doi:10.1001/jamanetworkopen.2025.44535

eMethods 1. Guidance for Safe Gait

eMethods 2. Criteria for Determining the Most Appropriate Walking Aid

eResults. Secondary Outcomes: Functional Capacity, Gait Assessment, Quality of Life, Cognition, Depression, and Occurrence of Falls

eTable 1. Quality of Life According to Levels and Dimensions of EQ-5D-3L

eTable 2. Occurrence of Falls at 90 Days

This supplementary material has been provided by the authors to give readers additional information about their work.

© 2025 Polesel FS et al. *JAMA Network Open*.

eMethods 1. Guidance for Safe Gait

These guidelines complement the information presented in the main article and aim to provide patients and caregivers with practical tips to promote safety and independence during gait.

Guidance for a Safe Gait

1. Stand up

- To get out of bed, first roll the body, flex the knees and put the legs out of the bed. Stand up with the support of arms and support tightly the feet on the floor. Wait a few minutes to prevent dizziness and imbalance.
- To stand up, support tightly both feet on the floor, slightly apart. Lean the torso forward. If necessary, use the support of your arms, the walking aid (cane or walker), or someone's help.
- Observe the stability on standing position; only if you stay steady with legs, torso and hip, without dizziness, you can start walk.

2. Walk

- Use supports, such as walking aids (cane or walker), another person or bar if necessary. Talk with your physician and/or physical therapist about walking aid indication to give you more safety and independence to walk.
- Try to walk slowly to better control your steps.
- Prefer comfortable and safe shoes that stick to the foot, avoiding slippers, very wide shoes, socks, high heels and shoes with slippery sole.
- Wear glasses and hearing aid if necessary. Keep glasses and/or hearing aids well adapted (regularly visit the ophthalmologist and/or otorhinolaryngologist).

© 2025 Polesel FS et al. *JAMA Network Open*.

- Avoid leaving objects in circulations areas that could cause an accident, such as rug and exposed wires, because of the risk of falling. Always keep the environment well lit.
- Walking time: according to medical advice. If you couldn't walk long distances, walk short distances more times a day.
- Always observe signs of tiredness (breathless, muscle or bone pain, lower limb tremors, intense sweating and excessive increase in heart rate) or imbalance. If you present one or more of these signs, interrupt the walk and rest. If the signs persist, contact your physician.

3. Go up and down the stairs

- Always use the stairs' handrails.
- Avoid narrow steps.
- Go up and down one step at a time.
- To go up the stairs, start by putting the foot of the strongest leg on the step and then the other foot, supporting both feet on the same step.
- To go down the stairs, start by putting the weakest leg on the step and then the other leg on the same step, going down one step at a time.

© 2025 Polesel FS et al. *JAMA Network Open*.

eMethods 2. Criteria for Determining the Most Appropriate Walking Aid

This section provides additional information on the criteria used to select the most appropriate walking aid for each patient, based on their individual needs and characteristics.

- **Canes:** one upper limb used for a walk, light weight-bearing and need for somatosensorial feedback; the cane must be positioned between 15 and 20 centimeters laterally to the feet; the patient's hand should be supported on the cane at the height of the greater trochanter of the femur and the elbow should be flexed approximately by 30°; in general, the cane is used on the opposite side of the injured leg.

- **Walkers:** both upper limbs used to walk, heavy weight bearing, and presence of postural instability; the equipment must be held between 20 and 25 centimeters in front of the body with relaxed shoulders, erect torso, and elbow flexed at 20° to 30°; during the total weight-bearing gait, the walker should be lifted and move forward approximately arm's length, while one lower limb move forward followed by the other, and the cycle repeats; in a non-weight bearing gait, the walker is lifted and moves forward, then the weight is transferred to the walker through the upper limbs, the affected limb is held in a position anterior to the person's body, but does not contact the ground, the unaffected limb is moved forward and this cycle repeats.

© 2025 Polesel FS et al. *JAMA Network Open*.

eResults. Secondary outcomes: functional capacity (Brathel Index and Lawton and Brody Scale), gait assessment (TUG test), quality of life (EQ-5D-3L), cognition (10-CS), depression (GDS-15), and occurrence of falls.

Functional capacity

Regarding Barthel Index scores, WA group exhibited significantly higher scores at 90 days compared with control group (MD: 8.93; 95% CI, 2.54 to 15.33; p= .007) and WAT group (MD: 7.92; 95% CI, 1.43 to 14.42; p= .02) (**Table 2**). Within-group analyses showed significant improvements in WA group (MD: 9.73; 95% CI, 1.92 to 17.54; p= .02) and WAT group (MD: 6.39; 95% CI, 2.99 to 9.82; p= .002), whereas control group experienced a significant decline over the same period (MD: -3.33; 95% CI, -6.40 to -0.25; p= .03) (**Table 3**).

For Lawton and Brody Scale scores, no significant differences were observed at 90 days between WA group and control group (MD: 1.67; 95% CI, -1.00 to 4.34; p= .21) or between WAT group and WA group (MD: 0.52; 95% CI, -2.01 to 3.05; p= .68) (**Table 2**). Within-group analyses revealed significant improvements in WA group (MD: 1.55; 95% CI, 0.08 to 3.02; p= .03) and WAT group (MD: 1.46; 95% CI, 0.07 to 2.85; p= .008), while no significant change was observed in control group (MD: 1.06; 95% CI, -2.84 to 4.96; p= .57) (**Table 3**).

Gait assessment

Immediately after the intervention, WA group showed a significant reduction in Timed Up and Go (TUG) test time compared with control group (MD: -10.45 seconds; 95% CI, -22.70 to -1.80; p= .02), with no difference versus WAT group (MD: -0.20 seconds; 95% CI, -12.97 to 12.57; p= .97) (**Table 2**). Within-

© 2025 Polesel FS et al. *JAMA Network Open*.

group analyses demonstrated significant reductions from baseline to postintervention in WA group (MD: -5.26 seconds; 95% CI, -9.16 to -1.36; p= .01) and WAT group (MD: -7.06 seconds; 95% CI, -12.64 to -1.47; p= .01). No significant change was observed in control group (MD, 6.47 seconds; 95% CI, -6.72 to 19.67; p= .31) (**Table 3**).

Quality of life

For quality-of-life scores, no significant differences were observed at 90 days between the WA group and control group (MD: -1.17; 95% CI, -7.34 to 4.99; p= .70) or between the WAT group and WA group (MD: 5.24; 95% CI, -0.82 to 11.31; p= .08) (**Table 2**). Within-group analyses showed significant improvements from baseline to 90 days in both WA group (MD: 10.66; 95% CI, 4.13 to 17.20; P = .002) and WAT group (MD, 11.06; 95% CI, 5.83 to 16.30; p< .001), whereas no significant change was observed in control group (MD: -3.21; 95% CI, -9.68 to 3.25; p= .30) (**Table 3**).

An analysis of the EQ-5D-3L dimensions for each group at baseline and 90 days can be found in **eTable 1**. Compared to baseline, at 90 days the control group showed a decline in self-care (level 1: 92% vs 61%; level 2: 8% vs 34%; p<0,05) and usual activity (level 1: 80% vs 61%; level: 2: 16% vs 34%; p<0,05) in levels 1 and 2 and mobility in the level 3 (0% vs 5%; p<0,05). WA group demonstrated an improvement in usual activity in the level 3 (16% vs 0%; p<0,05), but also an increase in anxiety/depression (0% vs 9%; p<0,05). While the WAT group did not show statistically significant improvements.

© 2025 Polesel FS et al. *JAMA Network Open*.

Cognition

At 90 days, the WA group demonstrated a significant increase in cognitive scores compared with the control group (MD: 1.74; 95% CI: 0.16 to 3.32; p= .03), with no significant difference versus WAT group (MD: 0.02; 95% CI: -1.52 to 1.57; p= .97) (**Table 2**). Within-group analyses showed significant increases from baseline to 90 days in both WA group (MD: 1.76; 95% CI: 0.84 to 2.68; p= .001) and WAT group (MD: 2.31; 95% CI: 0.59 to 4.02; p= .008), whereas no significant change was observed in control group (MD: -0.33; 95% CI: -2.08 to 1.42; p= .70) (**Table 3**).

Depression

For GDS-15 scores, no significant differences were observed at 90 days between the WA group and control group (MD: 0.02; 95% CI: -1.65 to 1.61; p= .98) or between the WAT group and WA group (MD: 0.40; 95% CI: -1.19 to 2.00; p= .61) (**Table 2**). Within-group analyses showed significant decreases from baseline to 90 days in both WA group (MD: -0.94; 95% CI: -1.24 to -0.65; p= .001) and WAT group (MD: -0.95; 95% CI: -1.30 to -0.60; p< .001), whereas no significant change was observed in control group (MD: -0.33; 95% CI: -1.83 to 1.16; p= .65) (**Table 3**).

Occurrence of falls

eTable 2 describes the number of falls, along with their severity and intensity. Within 90 days, three participants in the WA and WAT groups experienced falls, compared to four in the control group. All falls were classified

© 2025 Polesel FS et al. *JAMA Network Open*.

as non-serious. Additionally, the occurrence of falls was not associated with the provision of a walking aid.

© 2025 Polesel FS et al. *JAMA Network Open*.

eTable 1. Quality of Life According to Levels and Dimensions of EQ-5D-3L

		Baseline					90 days				
**Group**	**Level**	**Mobility n (%)**	**Self-care n (%)**	**Usual activity n (%)**	**Pain/Discomfort n (%)**	**Anxiety/Depression n (%)**	**Mobility n (%)**	**Self-care n (%)**	**Usual activity n (%)**	**Pain/Discomfort n (%)**	**Anxiety/Depression n (%)**
**WA Baseline (n=25) 90d (n=22)**	Level 1	15 (60)	19 (76)	16 (64)	7 (28)	16 (64)	11 (50)	16 (73)	13 (59)	10 (45)	8 (36)
	Level 2	10 (40)	5 (20)	5 (20)	17 (68)	9 (36)	11 (50)	6 (27)	9 (41)	12 (55)	12 (55)
	Level 3	0 (0)	1 (4)	4 (16)	1 (4)	0 (0)	0 (0)	0 (0)	0 (0)	0 (0)	2 (9)
**WAT Baseline (n=25) 90d (n=19)**	Level 1	10 (40)	18 (72)	15 (60)	5 (20)	10 (40)	9 (47)	12 (63)	11 (58)	7 (37)	11 (58)
	Level 2	15 (60)	7 (28)	9 (36)	19 (76)	14 (56)	10 (53)	7 (37)	8 (42)	12 (63)	8 (42)
	Level 3	0 (0)	0 (0)	1 (4)	1 (4)	1 (4)	0 (0)	0 (0)	0 (0)	0 (0)	0 (0)
**Control Baseline (n=25) 90d (n=18)**	Level 1	17 (68)	23 (92)	20 (80)	8 (32)	10 (40)	10 (56)	11 (61)	11 (61)	6 (34)	10 (56)
	Level 2	8 (32)	2 (8)	4 (16)	15 (60)	14 (56)	7 (39)	6 (34)	6 (34)	10 (56)	8 (44)
	Level 3	0 (0)	0 (0)	1 (4)	2 (8)	1 (4)	1 (5)	1 (5)	1 (5)	2 (10)	0 (0)

Note: Control = Control Group; WA = Walking Aid Group; WAT = Walking Aid and Training Group.

© 2025 Polesel FS et al. *JAMA Network Open*.

eTable 2. Occurrence of Falls at 90 Days

	WA	WAT	Control
**Falls (n)**	3	3	4
**Severity, n (%)**			
Not serious	3 (100%)	3 (100%)	4 (100%)
**Intensity**			
Mild	1 (3,33%)	3 (100%)	3 (75%)
Moderate	1 (3,33%)	0 (0%)	1 (25%)
Complete commitment	1 (3,33%)	0 (0%)	0 (0%)

Note: Control = Control Group; WA = Walking Aid Group; WAT = Walking Aid and Training Group.

© 2025 Polesel FS et al. *JAMA Network Open*.
