# Supplementary material for: Walking Aids and Locomotion Training in the Emergency Department: A Randomized Clinical Trial
Source: JAMA Netw Open. 2025 Nov 21;8(11):e2544535. doi: 10.1001/jamanetworkopen.2025.44535 (PMC12639483; doi:10.1001/jamanetworkopen.2025.44535)
Supplement: Supplement 3. — Data Sharing Statement [file jamanetwopen-e2544535-s003.pdf]

Data Sharing Statement

Polesel. Walking Aids and Locomotion Training in the Emergency Department. *JAMA Netw Open*. Published November 21, 2025. doi:10.1001/jamanetworkopen.2025.44535

Data
Additional Information: NCT05950269
Data available: No
